# Implementation of Medication-Related Technology and Its Impact on Pharmacy Workflow: Real-World Evidence Usability Study

**DOI:** 10.2196/59220

**Published:** 2025-03-27

**Authors:** Wei-Ning Yu, Yih-Dih Cheng, Yu-Chi Hou, Yow-Wen Hsieh

**Affiliations:** 1 Department of Pharmacy China Medical University Hospital Taichung City Taiwan; 2 College of Pharmacy, School of Pharmacy China Medical University Taichung City Taiwan

**Keywords:** medication error, dispensing error, medication-related technology, pharmacy, smart dispensing counter

## Abstract

**Background:**

Medication errors constitute a major contributor to patient harm, driving up health care costs and representing a preventable cause of medical incidents. Over the past decade, many hospitals have integrated various medication-related technologies into their pharmacy operations. However, real-world evidence of the impact of these advanced systems on clinical prescription dispensing error rates remains limited.

**Objective:**

This study aims to prospectively detect and record the categories and rates of dispensing errors to illustrate how medication-related technologies, such as automated dispensing cabinet (ADC), barcode medication administration (BCMA), and smart dispensing counter (SDC), can be used to minimize dispensing errors.

**Methods:**

This study used a before-and-after design at a 2202-bed academic medical center in Taiwan to assess the impact of implementing medication-related technologies (ADC, BCMA, and SDC) on patient medication safety. Dispensing error rates were analyzed from January 1, 2017, to December 31, 2023, using data from the China Medical University Hospital Patient Safety Database. The study periods were defined as stage 0 (preintervention, January to November 2017), stage 1 (post-ADC intervention, December 2017 to June 2018), stage 2 (post-BCMA intervention, July 2018 to October 2020), and stage 3 (post-SDC intervention, November 2020 to December 2023). Medication errors were defined according to the National Coordinating Council for Medication Error Reporting and Prevention (NCC MERP). Chi-square or Fisher exact tests were used to analyze differences between intervention periods, with Bonferroni correction for multiple comparisons. Statistical significance was set at *P*<.05.

**Results:**

Following the introduction of medication-related technologies, the average dispensing error incidence rate significantly decreased by 39.68%, 44.44%, and 77.78%, from 0.0063% in stage 0 to 0.0038%, 0.0035%, and 0.0014% in stages 1, 2, and 3, respectively (*P*<.001). The frequency of “wrong drug” errors, the most common error type in stage 0, significantly decreased by 51.15%, 56.85%, and 81.26% in stages 1, 2, and 3, respectively. All error types, except for “wrong dosage form,” “wrong strength,” “wrong time,” and “others,” demonstrated statistically significant differences (*P*<.001). The majority of harm severities were categorized as “A” (no error; 97%-98.8%) and “B-D” (error, no harm; 1.2%-3%) according to the NCC MERP classification. The severity of “no error” (category A) significantly decreased at each stage (*P*<.001). Statistically significant differences in dispensing error rates were observed between all stages (*P*<.001), except between stages 2 and 1 (*P*>.99).

**Conclusions:**

This study provides significant evidence that the implementation of medication-related technologies, including ADC, BCMA, and SDC, effectively reduces dispensing errors in a hospital pharmacy setting. Specifically, we observed a substantial decrease in the average dispensing error rate across 3 stages of technology implementation. Importantly, this study appears to be the first to investigate the combined impact of these 3 specific technologies on dispensing error rates within a hospital pharmacy.

## Introduction

Medication errors pose a significant risk to patient safety and incur substantial economic losses. Recognizing this, medication safety has been identified as a critical area for improvement in all health care settings globally [1]. In March 2017, the World Health Organization (WHO) launched the third Global Patient Safety Challenge, focusing specifically on medication safety. This initiative aims to address systemic weaknesses within health care systems that contribute to medication errors and their potentially severe consequences. The WHO has set an ambitious goal to globally reduce the level of severe, avoidable medication-related harm by 50% within 5 years. This challenge emphasizes improvements across all stages of the medication process, including prescribing, dispensing, administering, monitoring, and patient use [2,3]. In the United States, the Joint Commission on Accreditation of Healthcare Organizations also prioritizes medication safety by including it among its annual patient safety goals [4]. Previous research indicates that approximately 22%-25% of medication errors occur during the dispensing phase [5]. Dispensing errors often precede administration errors, highlighting the critical role of the pharmacy in preventing medication errors. Estimates of medication errors vary significantly across different regions within the prescription (10%-39%), dispensing (11%-40%), and administration (10.5%-38%) stages of medication management. These variations can be attributed to multiple factors [6-14].

Medication errors can arise when weak medication safety systems and human factors, such as fatigue, poor environmental conditions, or staff shortages, compromise any stage of the medication management process [15]. Dispensing errors can lead to preventable patient harm, including adverse drug events, hospitalization, or even death. A systematic review examining the global prevalence of dispensing errors across various pharmacy settings revealed rates ranging from 0% to 33.3%. The worldwide prevalence of dispensing errors was determined to be 1.6% across community, hospital, and other pharmacy settings [16]. Factors influencing dispensing errors include pharmacists’ machine usage, workload, and the length of their monthly vacations. Adequate staffing and the use of automated medication preparation systems may be necessary to minimize dispensing errors. Pharmacists returning to dispensing duties after an absence exceeding 72 hours should gradually reintegrate to regain their proficiency [17]. Near-misses, incidents with the potential for medication errors, often share the same root causes as actual errors [18]. Proactively identifying and addressing near-misses is crucial for preventing future medication errors.

Health information technology is believed to be transformative in addressing inefficiencies, preventing medication errors, and improving overall care quality [19]. The integration of health information technology throughout the medication use process is expected to enhance health care safety and efficiency. Past research demonstrates that automated equipment and systems are generally more effective than manual methods, providing a more standardized and reliable execution of medication use processes and thereby reducing errors [20-22].

The use of medication-related technologies, such as automated dispensing cabinets (ADCs), barcode medication administration (BCMA), and smart dispensing counters (SDCs), is increasingly prevalent in hospitals. These technologies aim to reduce medication errors, safeguard medications from improper use, and improve the efficiency of medication processes. This study aims to evaluate the impact of medication-related technologies on both workflow efficiency and prescription dispensing accuracy following their implementation in a Taiwan academic medical center.

## Methods

### Definitions of Medication Error

The definition of medication error mainly refers to the definition provided by the US National Coordinating Council for Medication Error Reporting and Prevention (NCC MERP). A medication error is defined as “any preventable event that may cause or lead to inappropriate medication use or patient harm while the medication is in the control of the health care professional, patient, or consumer” [3]. Medication errors occur at each stage of the medication process, including prescribing, dispensing, administering, monitoring, and use.

Dispensing medication is the core function of pharmaceutical care and involves a complex combination of processes, technologies, and human interactions. Dispensing errors include, but are not limited to, dispensing the medicine for the wrong patient, incorrect medicine name, incorrect strength, and incorrect dosage [7].

### Study Design

Our hospital experiences 190,000 outpatient visits; 12,000 emergency visits; 7600 inpatient admissions; and 5100 surgeries monthly. To evaluate the impact of implementing medication-related technologies (ADCs, BCMAs, and SDCs) on patient medication safety, we conducted a before-and-after study at our 2202-bed academic medical center in Taiwan.

We analyzed medication error rates within the medication use process from January 1, 2017, to December 31, 2023, using data from the China Medical University Hospital Patient Safety Database (Multimedia Appendices 1 and 2). Our analysis specifically focused on medication errors that occurred during pharmacist medication dispensing. Power business intelligence was used for visual data analytics to conduct a frequency and cause analysis of these errors.

### Intervention-Medication–Related Technologies

To address common errors in pharmacist medication dispensing, we used ADC, BCMA, and SDC as interventions. The study periods were defined as follows: stage 0 (preintervention, January to November 2017), stage 1 (post-ADC intervention, December 2017 to June 2018), stage 2 (post-BCMA intervention, July 2018 to October 2020), and stage 3 (post-SDC intervention, November 2020 to December 2023).

#### ADC Intervention

An ADC, also called a unit-based cabinet, automated dispensing device, or automated dispensing machine, is a computerized medicine cabinet for hospitals and health care settings. ADCs allow medications to be stored and dispensed near the point of care while controlling and tracking drug distribution [20].

#### BCMA Intervention

BCMA is a barcode system designed to prevent medication errors in health care settings and improve the quality and safety of medication administration. The overall goals of BCMA are to improve accuracy, prevent errors, and generate digital records of medication administration [20,22,23].

#### SDC Intervention

The SDC is also known as the LED-guided picking plus lockable drawer (LED-LD) system. The LED-LD system is linked to the medicine management information system, allowing the extraction of data on the location of the medication. The LED-LD system has adopted LED technology to aid in locating the correct medication bin. When scanning the quick response code on a medication label, the LED corresponding to the medication location lights up, directing the pharmacy staff to the correct medication bin. The LED-LD system uses a remote lockable drawer system integrated with LED technology. Upon scanning the quick response code, only the drawer containing the corresponding medication is unlocked, enabling its retrieval. The drawer locks automatically when it is shut [24] (Multimedia Appendix 3).

### Outcome

All medication orders, including those written or cosigned by physicians during the study period, were included in the analysis. The pharmacy, providing continuous services for both outpatients and inpatients, adheres to standardized protocols for medication preparation and dispensing. The primary outcome measure was the monthly rate of prescription dispensing errors before and after the implementation of medication-related technologies (ADC, BCMA, and SDC). This rate was calculated by dividing the number of reported prescription dispensing errors by the total number of prescriptions filled each month. Secondary outcome measures included the types of dispensing errors and their severity. The severity of medication errors was assessed by two pharmacists using the NCC MERP method. In case of disagreement, consensus was reached through discussion with a third senior pharmacist.

### Statistical Analysis

Descriptive statistics were used to summarize the dispensing error rates at each stage of the medication use process. The dispensing error rate was calculated by dividing the total number of dispensing errors by the total number of medications dispensed. We compared the incidence of dispensing errors before and after the implementation of medication-related technologies (ADCs, BCMAs, and SDCs). Furthermore, we stratified the analyses by the type and severity of dispensing errors.

Differences among the intervention periods were analyzed using chi-square or Fisher exact tests. The Bonferroni method was applied to adjust for multiple comparisons. All statistical analyses were conducted using R software (version 4.1.0, R Foundation for Statistical Computing). Statistical significance was defined as *P*<.05.

### Ethical Considerations

This study was conducted in accordance with the Declaration of Helsinki and approved by the Institutional Review Board of the Research Ethics Committee at China Medical University Hospital, Taichung, Taiwan (approval CMUH114-REC2-041). Following the 'Patient Safety Incident Reporting and Reward Operation Guidelines' of China Medical University Hospital, reporter information is automatically identified and transferred upon acceptance and establishment of a patient safety incident report, facilitating subsequent review and collaborative processes. This study utilized secondary use data, derived from a descriptive qualitative analysis of the patient safety notification database, to quantify error type categories related to healthcare information technologies. Notably, this study did not involve any patient or staff identification, and therefore, participant compensation disclosure is not applicable.

## Results

### Characteristics of Prescription Dispensing Errors of Prevalence, Type, and Potential Severity

Table 1 presents the proportions of prescription dispensing errors by prevalence, type, and potential severity before and after the implementation of medication-related technologies. A total of 15,410,968 medication orders were dispensed in stage 0 (preintervention), followed by 10,721,238 in stage 1; 44,193,666 in stage 2; and 56,260,136 in stage 3. The total number of dispensing errors observed were 968, 406, 1556, and 773 in stages 0, 1, 2, and 3, respectively. The average number of dispensing errors per month decreased from 88 in stage 0 to 58 in stage 1, 56 in stage 2, and 20 in stage 3. Correspondingly, the dispensing error rates declined from 0.0063% in stage 0 to 0.0038% in stage 1, 0.0035% in stage 2, and 0.0014% in stage 3.

In stage 0 (preintervention), the 3 most frequent types of dispensing errors were wrong drug (459/968, 47.4%), wrong dose (335/968, 34.6%), and wrong technique (55/968, 5.7%). While the specific ranks varied slightly across stages, wrong drug, wrong dose, and wrong patient consistently ranked among the top 3 types of dispensing errors. According to the NCC MERP classification, the severity of harm (97%-98.8%) was categorized as “A” (no error) in all stages. The remaining severity of harm primarily fell into categories “B-D” (error, no harm). In stage 2, two cases were categorized as “C” (dose omission and wrong dose), and one case was categorized as “D” (wrong drug), all with no harm. In stage 3, only one case of “C” (wrong dose) was observed, again with no harm.

**Table 1:** Characteristics of prescription dispensing error and the severity of harm before and after the implementation of medication-related technology.

Stage			Stage 0		Stage 1		Stage 2		Stage 3
Time periods			Preintervention (January to November 2017)		Post-ADC^a^ intervention (December 2017 to June 2018)		Post-BCMA^b^ intervention (July 2018 to October 2020)		Post-SDC^c^ intervention (November 2020 to December 2023)
Months, n			11		7		28		38
Total medication orders per prescription dispensed, n			15,410,968		10,721,238		44,193,666		56,260,136
Total dispensing errors, n			968		406		1556		773
Average dispensing errors per month, n			88		58		56		20
Dispensing error rate (%)^d^			0.0063		0.0038		0.0035		0.0014
**Types of dispensing errors, n (%)^e^**									
	Wrong drug	459 (47.4)		156 (38.4)		568 (36.5)		314 (40.6)	
	Wrong dose	335 (34.6)		133 (32.8)		549 (35.3)		216 (27.9)	
	Wrong dosage form	9 (0.9)		13 (3.2)		63 (4.0)		15 (1.9)	
	Wrong strength	12 (1.2)		4 (1.0)		58 (3.7)		35 (4.5)	
	Wrong patient	42 (4.3)		33 (8.1)		149 (9.6)		62 (8.0)	
	Wrong time	0 (0)		1 (0.2)		2 (0.1)		1 (0.1)	
	Dose omission	23 (2.4)		10 (2.5)		22 (1.4)		22 (2.8)	
	Monitoring error	9 (0.9)		4 (1.0)		2 (0.1)		0 (0)	
	Wrong technique	55 (5.7)		28 (6.9)		52 (3.3)		54 (7.0)	
	Others	24 (2.5)		24 (5.9)		91 (5.8)		54 (7.0)	
**Severity of harm^f^, n (%)^e^**									
	Category A (no error)	939 (97)		401 (98.8)		1529 (98.3)		760 (98.3)	
	Category B (error, no harm)	29 (3)		5 (1.2)		24 (1.5)		12 (1.6)	
	Category C (error, no harm)	0 (0)		0 (0)		2 (0.1)		1 (0.1)	
	Category D (error, no harm)	0 (0)		0 (0)		1 (0.1)		0 (0)	
	Category E (error, harm)	0 (0)		0 (0)		0 (0)		0 (0)	
	Category F (error, harm)	0 (0)		0 (0)		0 (0)		0 (0)	
	Category G (error, harm)	0 (0)		0 (0)		0 (0)		0 (0)	
	Category H (error, harm)	0 (0)		0 (0)		0 (0)		0 (0)	
	Category I (error, death)	0 (0)		0 (0)		0 (0)		0 (0)	

^a^ADC: automated dispensing cabinet.

^b^BCMA: barcode medication administration.

^c^SDC: smart dispensing counter.

^d^The number of dispensing errors divided by the number of medication orders.

^e^Percentage are calculated using the number of dispensing errors as the denominator.

^f^National Coordinating Council for Medication Error Reporting and Prevention (NCC MERP) classification [3]: no error (category A); error, no harm (categories B-D); error, harm (categories E-H); and error, death (category I). A: circumstances or events that have the capacity to cause an error; B: an error occurred but it did not reach the patient. C: an error occurred that reached the patient but did not cause harm to the patient; D: an error occurred that reached the patient and required monitoring to confirm that it resulted in no harm to the patient, or if necessary, required intervention to preclude harm; E: an error occurred that may have contributed to or resulted in temporary harm to the patient and required intervention; F: an error occurred that may have contributed to or resulted in temporary harm to the patient and required initial or prolonged hospitalization; G: an error occurred that may have contributed to or resulted in permanent harm to the patient; H: an error occurred that required intervention necessary to sustain life; and I: an error occurred that may have contributed to or resulted in the patient’s death.

### Effect of Medication-Related Technology on Reducing Dispensing Errors

**Figure 1:**
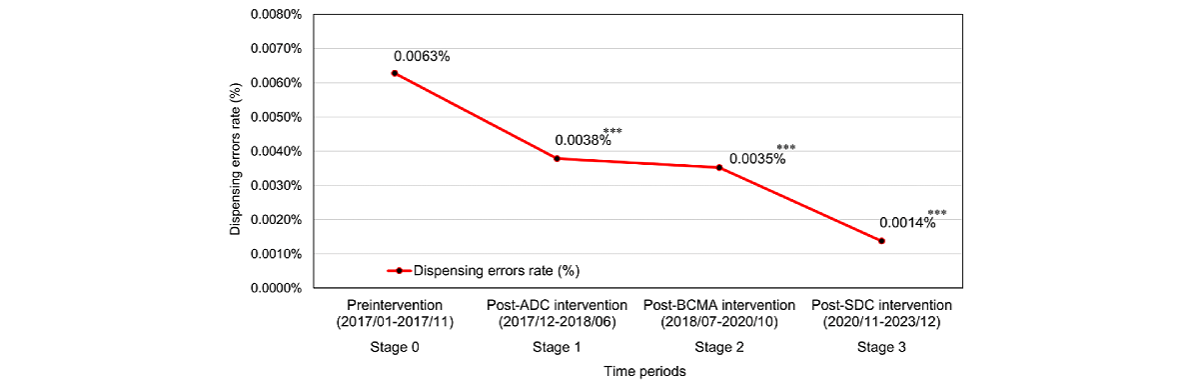
The trend curve of the dispensing error rate before and after the implementation of medication-related technology in each stage from 2017 to 2023. ***P<.001. ADC: automated dispensing cabinet; BCMA: barcode medication administration; SDC: smart dispensing counter.

Figure 1 shows a significant decrease in the rate of reported prescription dispensing errors across the observation periods. Compared with the preintervention stage 0 (traditional manual medication picking), where the average dispensing error rate was 0.0063%, a notable reduction was observed in subsequent stages: stage 1 (post-ADC intervention) at 0.0038% (39.68% decrease), stage 2 (post-BCMA intervention) at 0.0035% (44.44% decrease), and stage 3 (post-SDC intervention) at 0.0014% (77.78% decrease).

#### Effect of Introducing ADC on Reducing Dispensing Errors

Table S1 in Multimedia Appendix 4 shows the incidence of dispensing errors by type before and after the introduction of an ADC. Compared with the preintervention stage (stage 0), the post-ADC intervention stage (stage 1) demonstrated significant reductions in the incidence of the wrong drug (0.0030%-0.0015%; *P*<.001), wrong dose (0.0022%-0.0012%; *P*<.001), and total dispensing error rates (0.0063%-0.0038%; *P*<.001). Other error types, including wrong dosage form, wrong strength, wrong patient, wrong time, dose omission, monitoring error, wrong technique, and other errors, showed either minimal changes or slight decreases.

Regarding the severity of harm, a significant reduction was observed in category A (no error) from 0.0061% in stage 0 to 0.0037% in stage 1 (*P*<.001). Furthermore, the prevalence of category B (error, no harm) decreased from 0.00019% in stage 0 to 0.00005% in stage 1 (*P*=.02).

#### Effect of Introducing BCMA on Reducing Dispensing Errors

Table S1 in Multimedia Appendix 4 shows the incidence of dispensing errors by type before and after the introduction of BCMA. Compared with the preintervention stage (stage 0), the post-BCMA intervention stage (stage 2) demonstrated significant reductions in the incidence of wrong drug (0.0030%-0.0013%; *P*<.001), wrong dose (0.0022%-0.0012%; *P*<.001), dose omission (0.00015%-0.00005%; *P*=.001), monitoring errors (0.00006%-0.00001%; *P*<.001), wrong technique (0.0004%-0.0001%; *P*<.001), and the total dispensing error rate (0.0063%-0.0035%, *P*<.001). Compared with the post-ADC intervention stage (stage 1), only the wrong technique showed a significant reduction in stage 2 (*P*=.005).

Regarding the severity of harm, a significant reduction was observed in category A (no error) from 0.0061% in stage 0 to 0.0035% in stage 2 (*P*<.001). Similarly, category B (error, no harm) also demonstrated a significant reduction from 0.00019% in stage 0 to 0.00005% in stage 2 (*P*<.001). No significant differences in the severity of harm were observed between stage 1 and stage 2.

#### Effect of Introducing SDC on Reducing Dispensing Errors

Table S1 in Multimedia Appendix 4 shows the incidence of dispensing errors by type before and after the introduction of SDC. Compared with the preintervention stage (stage 0), the post-SDC intervention stage (stage 3) demonstrated significant reductions in the incidence of wrong drug (0.0030%-0.0006%; *P*<.001), wrong dose (0.0022%-0.0004%; *P*<.001), wrong patient (0.0003%-0.0001%; *P*<.001), dose omission (0.00015%-0.00004%; *P*<.001), monitoring errors (0.00006%-0%; *P*<.001), wrong technique (0.0004%-0.0001%; *P*<.001), and the total dispensing error rate (0.0063%-0.0014%; *P*<.001). Significant reductions were also observed between stage 1 and stage 2, as well as between stage 2 and stage 3 for multiple error types, including wrong drug, wrong dose, wrong dosage form, wrong patient, others, and the total dispensing error rate.

Regarding the severity of harm, a significant reduction was observed in category A (no error) from 0.0061% in stage 0 to 0.0014% in stage 3 (*P*<.001). Similarly, category B (error, no harm) also demonstrated a significant reduction from 0.00019% in stage 0 to 0.00002% in stage 3 (*P*<.001). Significant reductions in category A were also observed between stage 1 and stage 3 (*P*<.001), as well as between stage 2 and stage 3 (*P*<.001).

As given in Table 2, statistically significant reductions in prescription dispensing error rates were observed between each stage (*P*<.001), except when comparing stage 1 to stage 2.

**Table 2:** Comparisons of the prescription dispensing error rates at each stage.

Stage	Stage name	Prescribed medications, n	Dispensing errors, n (%)	*P* value	Comparison *P* value			
					Vs stage 0	Vs stage 1	Vs stage 2	Vs stage 3
0	Preintervention	15,410,968	968 (0.0063)	<.001^a^	—^b^	<.001^a^	<.001^a^	<.001^a^
1	Post-ADC^c^ intervention	10,721,238	406 (0.0038)	—	<.001^a^	—	>.99	<.001^a^
2	Post-BCMA^d^ intervention	44,193,666	1556 (0.0035)	—	<.001^a^	>.99	—	<.001^a^
3	Post-SDC^e^ intervention	56,260,136	773 (0.0014)	—	<.001^a^	<.001^a^	<.001^a^	—

^a^Statistically significant (*P*<.001) after adjustment using the Bonferroni correction.

^b^Not available.

^c^ADC: automated dispensing cabinet.

^d^BCMA: barcode medication administration.

^e^SDC: smart dispensing counter.

## Discussion

### Main Findings of Medication-Related Technology in Reducing Dispensing Errors

The study revealed that prescription dispensing error rates significantly decreased after the introduction of medication-related technologies, including ADC, BCMA, and SDC. Specifically, error rates reduced by 39.68%, 44.44%, and 77.78% from stage 0 (preintervention) to stage 1 (post-ADC intervention), stage 2 (post-BCMA intervention), and stage 3 (post-SDC intervention), respectively. The average number of dispensing errors per month also significantly decreased from 88 in stage 0 to 58 in stage 1, to 56 in stage 2, and to 20 in stage 3. The most frequent error type in stage 0, wrong drug, was significantly reduced by 51.15%, 56.85%, and 81.26% in stages 1 to 3, respectively. Similarly, the wrong dose, the second most frequent error type, was significantly reduced by 42.93%, 42.85%, and 82.34% across the stages. Compared with stage 0, the severity of harm, as categorized by category A (no error) and category B (error, no harm), was significantly decreased in all subsequent stages. These findings demonstrate the effectiveness of medication-related technologies in reducing dispensing errors in a hospital pharmacy and improving medication safety.

### The Actual Effectiveness of Introducing ADC in Reducing Dispensing Errors

In stage 1 (post-ADC intervention), the dispensing error rate decreased significantly from 0.0063% in stage 0 (preintervention) to 0.0038% (a reduction rate of 39.71%). This reduction was observed across all error types, including wrong drug, wrong dose, and the total number of dispensing errors. Notably, the reduction rate of wrong drug was substantial, decreasing from 0.0030% to 0.0015% (a 51.15% reduction), and the reduction rate of wrong dose also decreased significantly from 0.0022% to 0.0012% (a 42.93% reduction). These findings are consistent with previous studies that have demonstrated the effectiveness of ADCs in reducing dispensing errors [22,25-27]. The primary impact of ADC implementation was observed in a significant reduction of wrong drug and wrong dose. Previous research by Shah et al [26] highlighted that while ADCs can eliminate certain error types, they may also introduce new error types from a pharmacist’s perspective [26]. Therefore, pharmacists play a crucial role in ensuring the safe and effective use of ADCs by implementing appropriate policies, procedures, and quality assurance programs to address safety, accuracy, security, and patient confidentiality [28].

### The Actual Effectiveness of Introducing BCMA in Reducing Dispensing Errors

In stage 2 (post-BCMA intervention), the dispensing error rate decreased significantly by 43.95% compared with stage 0 (0.0063% vs 0.0035%). These findings were consistent across all error types, including wrong drug, wrong dose, monitoring error, wrong technique, dose omission, and the total number of dispensing errors. Notably, the reduction rate of the wrong drug was substantial, decreasing from 0.0030% to 0.0013% (56.85% reduction). The reduction rates of wrong dose, dose omission, and monitoring errors were also significant, decreasing from 0.0022% to 0.0012% (42.85% reduction), from 0.00015% to 0.00005% (66.67% reduction), and from 0.000058% to 0.000005% (92.25% reduction), respectively. The wrong technique error rate also decreased significantly from 0.0004% to 0.0001% (67.03% reduction). Compared with stage 1, only the wrong technique error rate showed a statistically significant difference. These findings are consistent with previous studies [29-31] and demonstrate the effectiveness of BCMA in reducing dispensing errors, particularly in preventing wrong drug, wrong dose, dose omission, monitoring error, and wrong technique.

Consistent with other studies, our analysis showed that BCMA implementation was associated with a reduction in dispensing errors, especially for medications with high error rates or those prone to confusion. However, the effectiveness of BCMA relies heavily on proper implementation, including comprehensive education and training on standard operating procedures. For example, simultaneous scanning of multiple medications can lead to errors, and issues with barcode quality or accuracy can also hinder the system’s effectiveness. In addition, damaged and unreadable drug barcodes, as well as medications labeled with incorrect barcodes, can also cause problems.

### The Actual Effectiveness of Introducing SDC in Reducing Dispensing Errors

In stage 3 (post-SDC intervention), the dispensing error rate decreased significantly by 78.13% compared with stage 0 (0.0063% vs 0.0014%). These findings were consistent across all error types, including wrong drug, wrong dose, wrong patient, dose omission, monitoring error, wrong technique, and the total number of errors. Notably, the reduction rate of wrong drug errors was substantial, decreasing from 0.0030% to 0.0006% (81.26% reduction). The reduction rates of wrong dose and wrong patient errors were also significant, decreasing from 0.0022% to 0.0004% (82.34% reduction) and from 0.0003% to 0.0001% (59.56% reduction), respectively. Monitoring errors were completely eliminated in stage 3. The wrong technique and dose omission error rates also decreased significantly. Compared with stage 1 and stage 2, significant reductions were observed in multiple error types in stage 3, including wrong drug, wrong dose, wrong patient, and others. The implementation of SDC demonstrated significant reductions in wrong drug, wrong dose, wrong patient, dose omission, monitoring error, and wrong technique. These findings are consistent with a previous study by Teo et al [24], which observed that technology-assisted medication picking systems, such as an LED-guided system with a lockable drawer, significantly reduced near-miss medication errors in a tertiary referral hospital in Singapore.

Our observations highlight the potential of the SDC system, designed to address the unique dispensing needs of Taiwan, to significantly reduce medication errors. By incorporating visual, auditory, and perceptual cues, the SDC system can guide pharmacists through the dispensing process, minimizing errors that may occur due to factors such as infrequent medication use, similar packaging, or distractions. This is particularly important for new pharmacists who are still familiarizing themselves with medication locations and workflow.

### Limitations

This study has several limitations. First, variations in medication error definitions across the literature make comparisons challenging. We attempted to standardize error classification according to the NCC MERP guidelines and calculated error rates based on the total number of dispensing errors and medication orders per prescription. Second, the staggered implementation of ADC, BCMA, and SDC (December 2017, July 2018, and November 2020, respectively) may have introduced a time-related bias. To mitigate this, we implemented standardized staff training on system operation and troubleshooting and provided on-site technical support. Furthermore, direct observation of drug dispensing was not conducted, making it difficult to confirm the absence of management errors despite random checks of standard dispensing procedures.

Automated systems may face challenges with irregularly shaped or infrequently used medications, requiring backup systems during downtime and maintenance [32]. Concurrent manual workflows during automation can also introduce potential sources of error. Based on our findings, optimizing the benefits of dispensing and management technologies requires adequate resource allocation for postimplementation support, including education, training, and technical assistance. The occurrence of system-related errors highlights the need for continuous monitoring and proactive problem-solving. For example, the system should track the movement of drugs after delivery, including any necessary withdrawals by pharmacists and nursing staff. Clear procedures for responding to system failures must be communicated to all employees. To maximize the benefits of these technologies, continuous evaluation, system refinement, and process optimization are crucial.

### Conclusions

This study demonstrated that the implementation of 3 medication-related technologies (ADC, BCMA, and SDC) significantly reduced dispensing errors compared with traditional manual medication picking. Overall, the study observed a significant reduction in dispensing errors across all stages. Except for wrong dosage form, wrong strength, wrong time, and other minor error types, all major error categories (including wrong drug, wrong dose, and wrong patient) showed statistically significant reductions (*P*<.001). The severity of harm also decreased significantly at each stage (*P*<.001). Significant differences in dispensing error rates were observed between all stages (*P*<.001). These findings underscore the potential of medication-related technologies to enhance medication safety and improve the efficiency of medication dispensing processes in hospital settings. Notably, the study observed significant reductions in critical error types such as wrong drug and wrong dose, which can have serious consequences for patient safety. This study shows that medication-related technology reduces dispensing errors and improves medication safety in hospitals. As hospitals work to enhance efficiency and safety, more targeted research is needed to assess the benefits and risks of these technologies. The findings highlight that technology can reduce errors and enhance medication management by improving dispensing and prescribing, optimizing clinical pharmacist resources, and allowing staff to focus more on patient care.

## Appendix

Multimedia Appendix 1 Types of dispensing error.

Multimedia Appendix 2 Severity of harm.

Multimedia Appendix 3 Smart dispensing counter (an LED-guided manual picking system) in a hospital pharmacy. LED-LD: LED-guided picking plus lockable drawer.

Multimedia Appendix 4 Table S1. Incidence of prescription dispensing errors and severity of harm before and after implementation of medication-related technology.

## Abbreviations

ADC: automated dispensing cabinet
BCMA: barcode medication administration
LED-LD: LED-guided picking plus lockable drawer
NCC MERP: National Coordinating Council for Medication Error Reporting and Prevention
SDC: smart dispensing counter
WHO: World Health Organization
