# Supplementary figures and images for: Implementation of Medication-Related Technology and Its Impact on Pharmacy Workflow: Real-World Evidence Usability Study
Source: J Med Internet Res. 2025 Mar 27;27:e59220. doi: 10.2196/59220 (PMC11986387; doi:10.2196/59220)

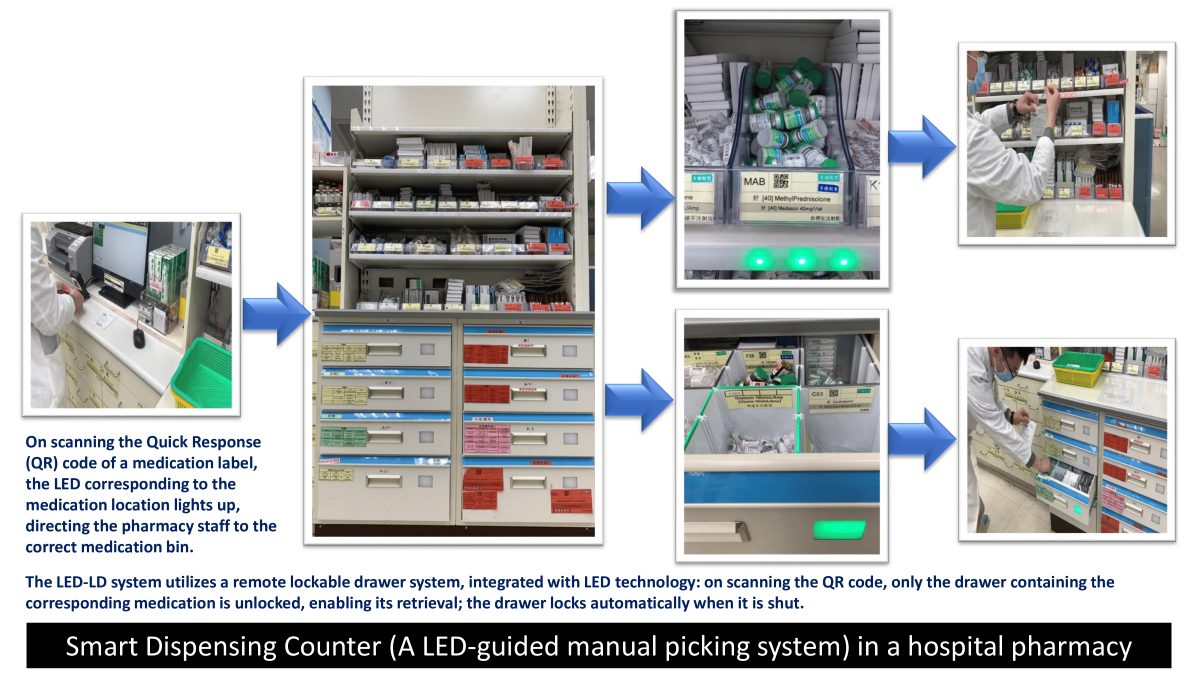

Supplement: Multimedia Appendix 3 [file jmir_v27i1e59220_app3.png]
