# Supplementary material for: Implementation of Medication-Related Technology and Its Impact on Pharmacy Workflow: Real-World Evidence Usability Study
Source: J Med Internet Res. 2025 Mar 27;27:e59220. doi: 10.2196/59220 (PMC11986387; doi:10.2196/59220)
Supplement: Multimedia Appendix 4 [file jmir_v27i1e59220_app4.docx]

**Table S1.** Incidence of prescription dispensing errors and severity of harm before and after implementation of medication-related technology.

| Stage | | Stage 0 | Stage 1 | | Stage 2 | | | Stage 3 | | | |
| --- | --- | --- | --- | --- | --- | --- | --- | --- | --- | --- | --- |
| Time periods | | Preintervention (January to November 2017) | Post-ADC^a^ intervention (December 2017 to June 2018) | | Post-BCMA^b^ intervention (July 2018 to October 2020) | | | Post-SDC^c^ intervention (November 2020 to December 2023) | | | |
|  |  | Number (%) | Number (%) | *P*-value^d^ vs Stage 0 | Number (%) | *P*-value^d^ vs Stage 0 | *P*-value^d^ vs Stage 1 | Number (%) | *P*-value^d^ vs Stage 0 | *P*-value^d^ vs Stage 1 | *P*-value^d^ vs Stage 2 |
| Prescribed medications | | 15,410,968 (100) | 10,721,238 (100) | —^e^ | 44,193,666 (100) | — | — | 56,260,136 (100) | — | — | — |
| **Type of dispensing error rate, n (%)^f^** | | | | | | | | | | | |
|  | Wrong Drug | 459 (0.0030) | 156 (0.0015) | <.001*** | 568 (0.0013) | <.001*** | >.99 | 314 (0.0006) | <.001*** | <.001*** | <.001*** |
|  | Wrong Dose | 335 (0.0022) | 133 (0.0012) | <.001*** | 549 (0.0012) | <.001*** | >.99 | 216 (0.0004) | <.001*** | <.001*** | <.001*** |
|  | Wrong Dosage Form | 9 (0.00006) | 13 (0.00012) | .79 | 63 (0.00014) | .08 | >.99 | 15 (0.00003) | .58 | <.001*** | <.001*** |
|  | Wrong Strength | 12 (0.00008) | 4 (0.00004) | >.99 | 58 (0.00013) | .75 | .09 | 35 (0.00006) | >.99 | >.99 | <.01** |
|  | Wrong Patient | 42 (0.0003) | 33 (0.0003) | >.99 | 149 (0.0003) | >.99 | >.99 | 62 (0.0001) | <.001*** | <.001*** | <.001*** |
|  | Wrong Time | 0 (0) | 1 (0.000009) | >.99 | 2 (0.000005) | >.99 | >.99 | 1 (0.000002) | >.99 | >.99 | >.99 |
|  | Dose Omission | 23 (0.00015) | 10 (0.00009) | >.99 | 22 (0.00005) | .001** | .88 | 22 (0.00004) | <.001*** | .21 | >.99 |
|  | Monitoring Error | 9 (0.000058) | 4 (0.000037) | >.99 | 2 (0.000005) | <.001*** | .10 | 0 (0) | <.001*** | <.001*** | >.99 |
|  | Wrong Technique | 55 (0.0004) | 28 (0.0003) | >.99 | 52 (0.0001) | <.001*** | .005** | 54 (0.0001) | <.001*** | <.001*** | >.99 |
|  | Others | 24 (0.0002) | 24 (0.0002) | >.99 | 91 (0.0002) | >.99 | >.99 | 54 (0.0001) | .38 | .004** | <.001*** |
|  | Total | 968 (0.0063) | 406 (0.0038) | <.001*** | 1556 (0.0035) | <.001*** | >.99 | 773 (0.0014) | <.001*** | <.001*** | <.001*** |
| **Severity of harm^g^, n (%)^f^** | | | | | | | | | | | |
|  | Category A (no error) | 939 (0.0061) | 401 (0.0037) | <.001*** | 1,529 (0.0035) | <.001*** | >.99 | 760 (0.0014) | <.001*** | <.001*** | <.001*** |
|  | Category B (error, no harm) | 29 (0.00019) | 5 (0.00005) | .02* | 24 (0.00005) | <.001*** | >.99 | 12 (0.00002) | <.001*** | >.99 | .06 |
|  | Category C (error, no harm) | 0 (0) | 0 (0) | — | 2 (0.000005) | >.99 | >.99 | 1 (0.000002) | >.99 | >.99 | >.99 |
|  | Category D (error, no harm) | 0 (0) | 0 (0) | — | 1 (0.000002) | >.99 | >.99 | 0 (0) | — | — | >.99 |
|  | Category E (error, harm) | 0 (0) | 0 (0) | — | 0 (0) | — | — | 0 (0) | — | — | — |
|  | Category F (error, harm) | 0 (0) | 0 (0) | — | 0 (0) | — | — | 0 (0) | — | — | — |
|  | Category G (error, harm) | 0 (0) | 0 (0) | — | 0 (0) | — |  | 0 (0) | — | — | — |
|  | Category H (error, harm) | 0 (0) | 0 (0) | — | 0 (0) | — | — | 0 (0) | — | — | — |
|  | Category I (error, death) | 0 (0) | 0 (0) | — | 0 (0) | — | — | 0 (0) | — | — | — |
| ^a^ADC: automated dispensing cabinet.  ^b^BCMA: barcode medication administration.  ^c^SDC: smart dispensing counter.  ^d^Statistically significant (****P*<.001; ***P*<.01; **P*<.05) after adjustment using the Bonferroni correction.  ^e^Not available.  ^f^The number of dispensing errors divided by the number of medication orders.  ^g^NCC MERP classification [3]: no error (category A); error, no harm (category B to D); error, harm (category E to H); and error, death (category I). A: circumstances or events that have the capacity to cause error; B: an error occurred but the error did not reach the patient. C: an error occurred that reached the patient but did not cause patient harm; D: an error occurred that reached the patient and required monitoring to confirm that it resulted in no harm to the patient, or if necessary, required intervention to preclude harm; E: an error occurred that may have contributed to or resulted in temporary harm to the patient and required intervention; F: an error occurred that may have contributed to or resulted in temporary harm to the patient and required initial or prolonged hospitalization; G: An error occurred that may have contributed to or resulted in permanent patient harm; H: an error occurred that required intervention necessary to sustain life; I: an error occurred that may have contributed to or resulted in the patient's death. | | | | | | | | | | | |
